# Sex differences in Alzheimer’s disease: a systematic review of two decades of neuroimaging research

**DOI:** 10.1093/bjr/tqag011

**Published:** 2026-01-16

**Authors:** Parinaz Massoumzadeh, Savannah Tiemann Powles, Mahshid Naghashzadeh, Jacqueline Rizzo, Jessica Hu, Lauren Yaeger, Hiba Alkelani, Qing Wang, Gengsheng Chen, Mahsa Dolatshahi, Nelly Joseph-Mathurin, Tammie L S Benzinger

**Affiliations:** Mallinckrodt Institute of Radiology, Washington University in St Louis, St Louis, MO 63110, United States; Mallinckrodt Institute of Radiology, Washington University in St Louis, St Louis, MO 63110, United States; Mallinckrodt Institute of Radiology, Washington University in St Louis, St Louis, MO 63110, United States; Mallinckrodt Institute of Radiology, Washington University in St Louis, St Louis, MO 63110, United States; Mallinckrodt Institute of Radiology, Washington University in St Louis, St Louis, MO 63110, United States; Mallinckrodt Institute of Radiology, Washington University in St Louis, St Louis, MO 63110, United States; Mallinckrodt Institute of Radiology, Washington University in St Louis, St Louis, MO 63110, United States; Mallinckrodt Institute of Radiology, Washington University in St Louis, St Louis, MO 63110, United States; Mallinckrodt Institute of Radiology, Washington University in St Louis, St Louis, MO 63110, United States; Mallinckrodt Institute of Radiology, Washington University in St Louis, St Louis, MO 63110, United States; Mallinckrodt Institute of Radiology, Washington University in St Louis, St Louis, MO 63110, United States; Mallinckrodt Institute of Radiology, Washington University in St Louis, St Louis, MO 63110, United States

**Keywords:** sex differences, Alzheimer’s disease, dementia, ADRD, MRI, amyloid-beta PET, tau-PET, neuroimaging, cognitive reserve

## Abstract

**Objectives:**

Given the heterogeneous nature of Alzheimer’s disease (AD) and its higher prevalence in females, it is crucial to understand sex-related differences in AD presentation and changes in the brain.

**Methods:**

This systematic review investigates sex differences in AD and summarizes key findings from neuroimaging studies over the past two decades to examine how genetics, hormones, and lifestyle factors influence neuroimaging biomarkers and their correlation with cognitive decline and AD progression. A comprehensive literature search was conducted across several databases for human studies from 2004 to 2024 related to AD, biological sex differences, and neuroimaging.

**Results:**

After a 3-step review process, the final extraction included 120 peer-reviewed studies using various neuroimaging modalities, such as MRI, amyloid-beta PET, tau-PET, and fluorodeoxyglucose (FDG) PET, to investigate sex as a biological predictor variable in adults with or at risk for AD. Over 90% of the reviewed studies identified clear sex-specific patterns of imaging biomarkers related to cognitive reserve, hormonal changes, *APOE-ɛ4* genotype, inflammation, vascular health, and lifestyle factors. Machine learning studies increasingly incorporate sex as a key variable, revealing sex-specific biomarkers and improving model performance in predicting disease status and progression.

**Conclusions:**

Considering biological sex in AD research is essential for improving diagnostic accuracy, tailoring interventions, and health outcomes.

**Advances in knowledge:**

This systematic review identifies sex-specific patterns in neuroimaging biomarkers of AD, influenced by cognitive reserve, hormones, APOE-ɛ4 genotype, inflammation, vascular health, and lifestyle. Recognizing these differences is crucial for understanding, diagnosis, and treatment efficacy.

## Introduction

Alzheimer’s disease (AD) is a neurodegenerative disorder characterized by progressive cognitive decline and memory loss that significantly reduce quality of life.^1–3^ Early detection and monitoring of AD are crucial for effective intervention.^3–6^ Neuroimaging techniques have become indispensable diagnostic and research tools for AD and can provide critical insights that are not achievable from clinical symptoms alone.^4^^,^^6–9^ Advanced imaging techniques, such as PET, MRI, and functional MRI (fMRI), enable the visualization and quantification of various biomarkers associated with AD, including amyloid plaques, tau tangles, and neurodegeneration.^2^^,^^5^^,^^6^^,^^8–10^ These imaging biomarkers are pivotal to understanding AD progression, facilitating early diagnosis, monitoring treatment responses, and tailoring personalized therapeutic approaches.^6^^,^^8^^,^^9^

Given the heterogeneous nature and varied presentations of AD, neuroimaging data can be used to understand the differences in AD presentation between males and females.^8^^,^^11^^,^^12^ Such biological differences play crucial roles in shaping health outcomes and disease patterns.^13^ Similarly, females are at higher risk for AD and comprise nearly two-thirds of all AD cases.^12–15^ To understand this, it is important to investigate sex differences in imaging biomarkers^8^^,^^13^ and how sex-specific risk factors, such as hormonal changes,^11^^,^^16^^,^^17^ genetic susceptibilities,^8^^,^^12^^,^^18^^,^^19^ and lifestyle factors,^20^ interact with these markers.^21^

A systematic review of neuroimaging-related sex differences in AD risk, diagnosis, presentation, and treatment is essential to characterize the unique factors that influence disease pathogenesis and progression, enhance diagnostic accuracy, tailor interventions more effectively, and guide future research to address current gaps. In this review, we summarize key findings from multiple neuroimaging studies and highlight how sex differences in imaging biomarkers are influenced by genetics, hormones, and lifestyle factors and their correlation with cognitive decline and AD progression. Ultimately, this will lead to improved health outcomes for all individuals affected by AD.

## Methods

### Literature search

All search strategies and methodologies were developed in alignment with the standards and guidelines for conducting and reporting systematic reviews outlined by the Preferred Reporting Items for Systematic Reviews and Meta-Analyses (PRISMA),^22^ and the Cochrane Handbook for Systematic Reviews.^23^

A medical librarian conducted a comprehensive literature search for human studies related to AD, biological sex differences, and neuroimaging from 2004 to 2024. The search strategies utilized a combination of keywords and were conducted across the following databases: Embase, Scopus, Ovid Medline, Cochrane Central Register of Controlled Trials and The Cochrane Database of Systematic Reviews, APA PsycInfo, and ClinicalTrials.gov. Fully reproducible search strategies for each database are detailed in Appendix S1.

### Exclusion and inclusion criteria

Peer-reviewed studies from 2004 to 2024 that investigated sex as a biological predictor variable of imaging biomarkers related to AD in adult human participants were eligible for inclusion in this review. Studies were excluded if they did not analyse sex as a biological predictor variable, did not discuss sex, did not analyse or compare imaging data, or did not address AD. Also, they were excluded if they were not published in English, were not peer-reviewed, or were non-human studies, review articles, case studies, or conference abstracts. Any additional studies matching all the exclusion and inclusion criteria that were identified outside of the systematic review were reviewed as a separate subset and added manually.

### Screening and data extraction

Independent reviewers employed the Covidence review management software (Veritas Health Innovation, Melbourne, Australia, www.covidence.org) to screen titles and abstracts according to the pre-specified inclusion and exclusion criteria. The full texts of studies that passed the initial screening were thoroughly reviewed, and ineligible studies were excluded. A comprehensive data extraction form was developed to extract information from all eligible studies on the type of dataset, study objectives, type of neuroimaging techniques, participant characteristics, factors of interest included in the models, and key findings related to sex differences. Additionally, to understand the landscape of the research, information was extracted on the institution, the country of data collection, the data centre’s website, and contact information. A second reviewer independently evaluated all studies that met the inclusion criteria and verified the data extraction files.

## Results

### Search and screening results

**Figure 1.**
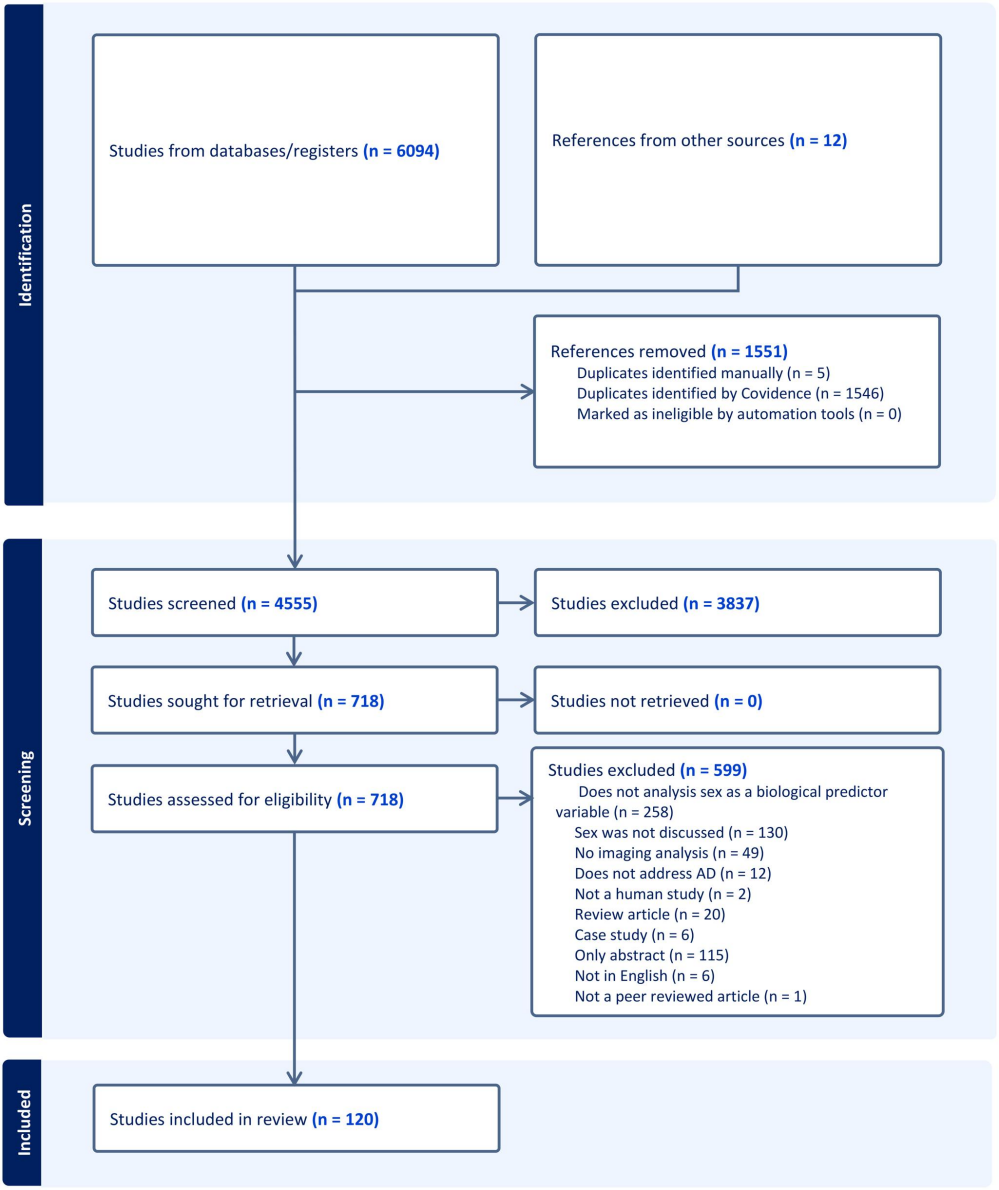
PRISMA chart outlining the selection of manuscripts included in this review.

Of the 6094 records initially identified, 1546 duplicate records were removed automatically, an additional 5 duplicates were identified and removed manually, and 12 studies were added manually. This resulted in 4555 unique records, of which 3837 citations were excluded during title and abstract screening. A full-text review was subsequently performed on the remaining 718 studies, of which 120 met the inclusion criteria (Figure 1).

**Figure 2.**
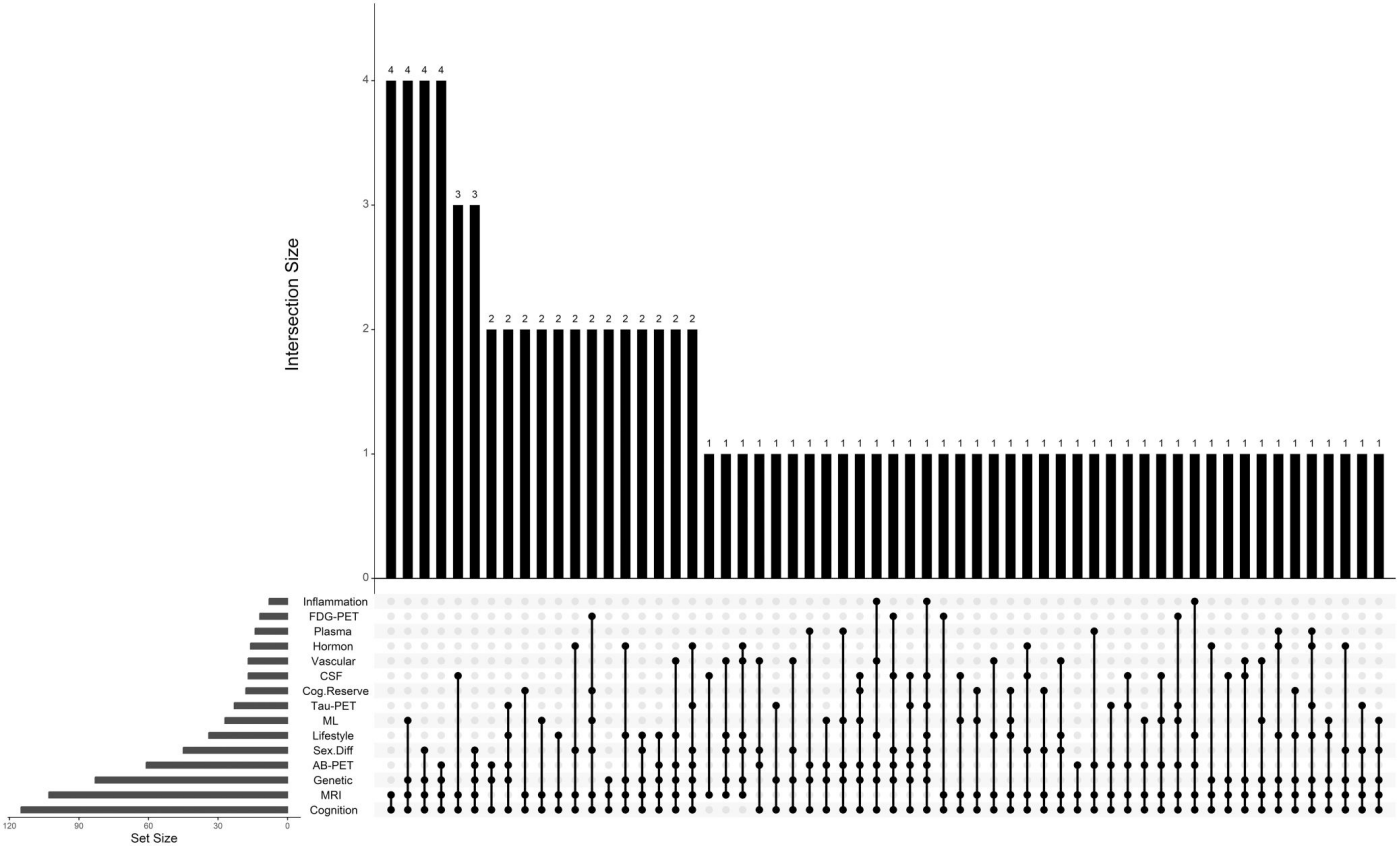
Intersection of data modalities across studies investigating sex differences in Alzheimer’s disease. This UpSet plot displays the combinations of imaging and biomarker modalities used in studies examining sex differences that are included in this review. Each dot and connecting line represents an intersection of modalities. The vertical bars indicate the number of studies using each combination. The horizontal bars on the left represent the total number of studies that are used in each individual modality, regardless of combination. The horizontal bars on the left represent the total number of studies that used each individual modality, regardless of combination. Abbreviations: Aβ-PET = amyloid beta PET; CSF, cerebrospinal fluid; Cog. reserve, cognitive reserve; FDG-PET, fluorodeoxyglucose PET; ML, machine learning; Sex diff. = primary studies of sex difference; Tau-PET = tau-PET.

**Figure 3.**
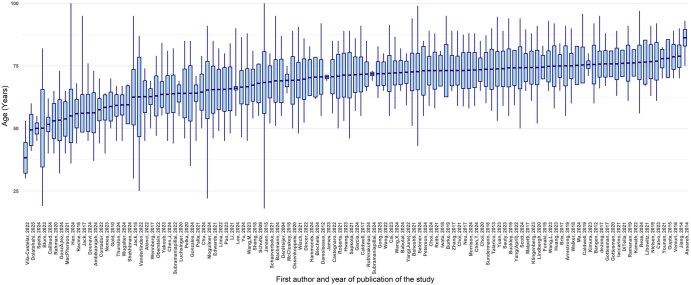
Distribution of participant ages across studies investigating sex differences in Alzheimer’s disease (AD). Each box represents a study included in this review. The centre line shows the mean participant age, while boxes show ±1 SD, and whiskers show the reported minimum and maximum ages in each study. Studies are ordered by descending mean age and labelled by first author and publication year.

**Figure 4.**
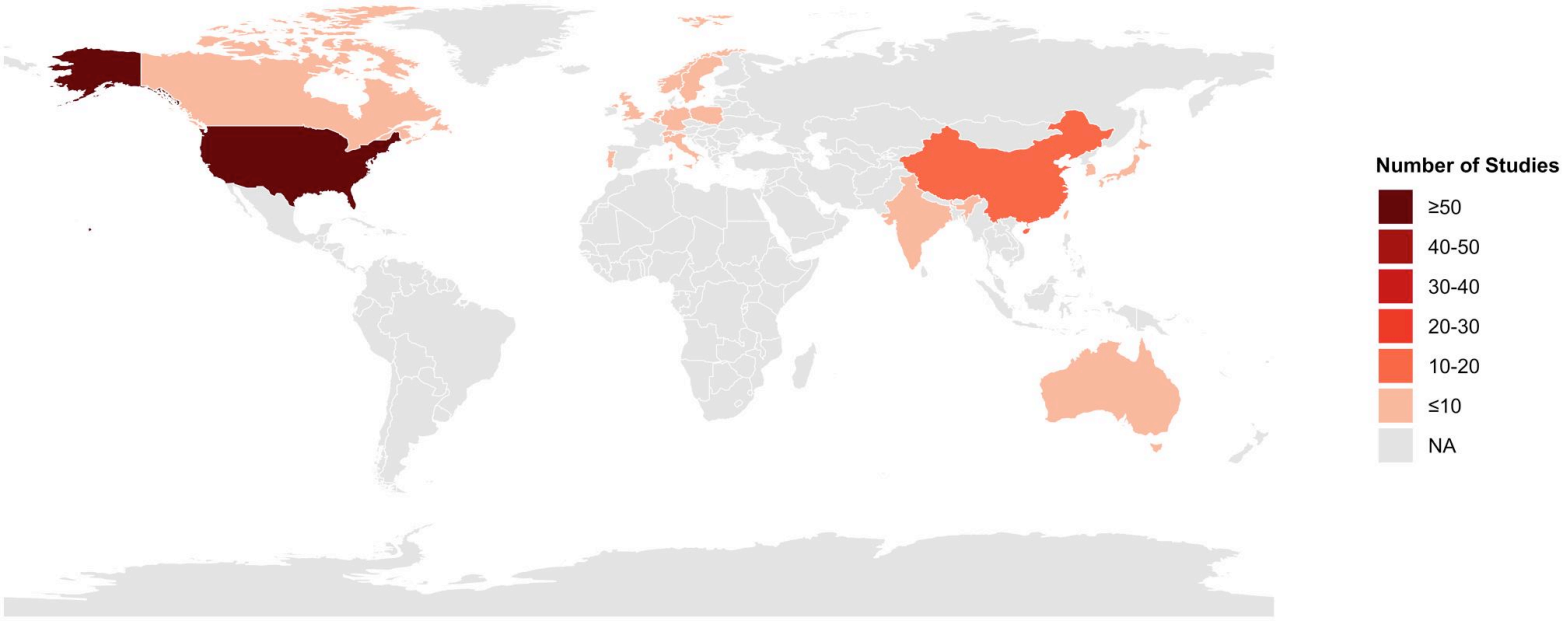
Distribution of countries of first authors’ institutional affiliations in studies investigating sex differences in Alzheimer’s disease (AD). This figure shows the number of studies that were conducted in each country, as determined by the institutional affiliations of first authors. The United States contributed the highest number of studies (*n* = 56), followed by China (*n* = 11). This systematic review includes only 2 multi-country/global analyses. Since this figure was created using the first authors’ institutional affiliation, it does not show countries included in global studies. One global analysis^72^ included 29 countries across North America, South America, Western Europe, Oceania, Eastern Europe, Asia, and Africa. Another global analysis^71^ included 15 countries in Latin America and the Caribbean, which are not depicted in this figure.

The primary objective of 45 of these 120 studies was to investigate sex differences in AD. The neuroimaging modalities used included MRI (*n* = 104), amyloid-beta PET (Aβ-PET) (*n* = 62), Tau-PET (*n* = 23), fluorodeoxyglucose (FDG)-PET (*n* = 12), and SPECT (*n* = 2). Additionally, some studies investigated sex interaction with other AD biomarkers and risk factors: cerebrospinal fluid (CSF) (*n* = 28), plasma (*n* = 14), apolipoprotein E ε4 (*APOE-ɛ4*, *n* = 83) and other genetic markers (*n* = 21), lifestyle (*n* = 35), hormone/menopause (*n* = 16), inflammation (*n* = 8), and vascular health (*n* = 17). Moreover, several studies used machine learning (*n* = 29). The breakdown of the studies is shown in Figure 2. Only 12 of the 120 studies did not identify significant sex-based differences in any of their variables of interest, including effects of bilingualism, sleep duration, and air pollution, but these variables were still found to be risk factors for AD.^18^^,^^24^^,^^25^  Table S1A provides a brief summary of all studies reviewed, including sex difference findings, number of participants, percentage of females, primary objective regarding sex differences, use of neuroimaging modalities (MRI, Aβ-PET, Tau-PET, FDG-PET), and genetic risk factors. Table S1B lists interactions with cognitive reserve, hormones, genetics, inflammation/vascular factors, lifestyle, machine learning and notes if no sex differences were found. Participants’ ages (mean, SD, minimum, maximum) across studies are plotted in Figure 3. Figure 4 shows the number of studies that were conducted in each country, as determined by the institutional affiliations of first authors, which also highlights collaborative efforts across multiple countries, accounting for approximately 28% of the publications. The United States was the leading contributor with 56 studies, followed by China with 11 studies. A breakdown of the exact number of publications from each country is included in Table S2. Notably, the chi-square goodness-of-fit test revealed that a significant number of studies were from regions with a majority non-Hispanic White population (*P* < .0001). A summary of public datasets utilized across publications is included in Table S3.

### Sex differences in cognitive reserve: implications for AD progression

The collective neuroimaging results from the included studies show that differences in cognition, and notably cognitive reserve, received research attention, appearing in hormone,^26^^,^^27^ machine learning,^19^^,^^28^^,^^29^ lifestyle,^18^^,^^24^^,^^26^^,^^30^ amyloid-PET,^26^^,^^28^^,^^31–35^ and MRI^36–38^ studies. Cognitive reserve reflects the brain’s ability to preserve cognitive functioning despite the development of AD-related pathology, such as the buildup of amyloid plaques and tau protein tangles. Table 1 provides a short summary of the manuscripts and findings related to cognitive reserve.

**Table 1.** Overview of manuscripts and key findings related to cognitive reserve.

**First author** **year**	***N*** **(F%)**	**Age** **mean**	MRI	Aβ- PET	Tau-PET	FDG-PET	Brief summary of manuscripts and sex-related findings
Ma 2024	1071 (46%)	75.2	1	1	1	1	Investigated sex-dependent effects of APOE; found sex moderates the rate of cognitive decline associated with APOE4 genotype.
Nemes 2023	377 (54%)	58.4	1	1	1		Used sex and APOE ε4 status as predictors of pathologic burden in early-onset Alzheimer’s disease; found sex-based effects on amyloid/tau-PET burden.
Ossenkoppele 2020	260 (56%)	69.2	1	1	1		Examined demographic, genetic, and imaging biomarker associations with brain resilience and tau burden; found that female sex is associated with resilience.
Vila-Castelar 2022	242 (62%)	38.3	1	1			Examined the effect of sex on pathology and memory in PSEN1 mutation carriers; found females exhibited better delayed recall than males.
Caldwell 2017	742 (48%)	71.6	1	1			Investigated the moderating effect of sex on amyloid and AD-related verbal learning deficits and hippocampal volume changes; sex differences were observed.
Wang, L. 2023	72 (69%)	74.8	1	1			Examined the role of age, sex, and education in association to WMH and Aβ deposition; found that sex is the strongest predictor of Aβ deposition.
Caldwell 2019	158 (50%)	75.4	1	1			Investigated whether sex moderated the effects of amyloid and APOE ε4 on default mode network connectivity; found sex-specific effects in females.
Lindbergh 2020	149 (52%)	74.4	1	1			Evaluated sex differences in β-amyloid and cognitive trajectories in preclinical AD; found that females with elevated Aβ are more vulnerable to memory decline.
Kimura 2023	122 (56%)	75.5	1	1			Predicted amyloid positivity using machine learning with wearable data; Sex is used as a predictor, and its significance varied across the models.
Beheshti 2021	1,280 (50%)	72.6	1				Explored sex differences in metabolic brain-age from fluorodeoxyglucose PET; found healthy females showed a younger metabolic brain age than males.
Malpetti 2017	507 (56%)	74.3	1				Examined sex differences in brain metabolic activity; found sex differences in correlations between education and brain hypometabolism.
Chen 2024	35,035 (53%)	63.6	1				Studied effects of age, APOE, and environment on the brain; found sex-specific APOE-ε4 effects on amygdala and cerebellum, plus age-sex interactions.
Li 2021	201 (58%)	65.8	1				Explored sex differences in brain network topology during AD; found sex differences in cognitive function and network architecture.
Calvo 2023	47 (49%)	76.9	1				Studied grey matter volume as a cognitive reserve indicator in bilinguals with MCI; found no significant sex-based differences.
DiTella 2021	82 (54%)	76.0	1				Identified neural predictors of the efficacy of multimodal rehabilitative AD interventions; found that females are more likely to improve in behavioural symptoms.
Jiang 2014	345 (52%)	78.9	1				Longitudinally studied the effects of sex and education on brain atrophy; found mixed significant sex effects in brain volumes and rate of atrophy.
Castegnaro 2022	70 (52%)	70.7	1				Assessed MCI using object-location memory; found that females made fewer location-binding errors than males.
Gustavsson 2023	4.E + 08 (54%)	Unstated					Investigated and provided global estimates of AD prevalence, stratified by sex; found a higher weighted global mean for women. Classifier
Jansen 2015	7583 (49%)	68.3					Conducted a meta-analysis on the prevalence of cerebral amyloid pathology; did not find significant associations between amyloid positivity and sex.

Abbreviations: N (F%) = number of participants and the percentage of female participants; Age mean = average age of participants in each study; MRI = magnetic resonance imaging; Aβ-PET = amyloid-beta PET; FDG-PET = fluorodeoxyglucose PET; Tau-PET = tau-PET.

### Interplay between sex differences and other factors

Table S4 shows the results of this systematic review, featuring a breakdown of the imaging biomarkers and variables of interest discussed in the studies reviewed. Binary notation indicates the variables included in each paper, alongside a summary of the study objective and primary results. This review showed that sex influences hormones and genetics, as well as inflammation, vascular health, and lifestyle as reported in some studies.

#### The impact of hormonal changes on neuroimaging finding

This systematic review included 16 studies with findings relevant to postmenopausal hormone therapy (MHT) or physiologic female-related hormone differences in menopausal stage, associated with neuroimaging biomarkers of AD. Sex differences were observed in all 16 studies, as summarized and presented in Table S5.

#### Genetics affecting AD pathology

As shown in Table S6, 85 studies discussed the relationship between genetics and neuroimaging biomarkers, including *APOE-ɛ4* carrier status. Genetic factors most commonly intersect with MRI and amyloid-PET scans (Figure 2). Sex differences were observed in 77 of the 85 studies.

#### Sex dependencies of inflammation and vascular health in AD

Table S7 displays 22 studies that discussed the relationship between inflammation and/or vascular health and neuroimaging biomarkers of AD, including white matter lesions or white matter hyperintensity, and intracranial atherosclerotic plaque on magnetic resonance angiogram. Inflammation and vascular health variables intersected with amyloid-PET, MRI, genetic risk factors (ie, *APOE-ɛ4*), and plasma biomarkers (Figure 2). Significant sex differences were found in 19 studies.

#### Lifestyle factors as AD risk factors

Thirty-five studies assessed variables related to individuals’ lifestyles, including body mass index (BMI), body adiposity, cardiovascular risk factors, education, sleep, systemic levels of inflammatory proteins, and air quality (Table S8). Studies featuring lifestyle factors heavily intersected with MRI findings (Figure 2). Sex differences were observed in 30 studies.

### Research avenues in the landscape of big data and machine learning

Table S9 shows 29 studies that utilized deep learning/machine learning as a key method when developing a model or examining sex differences in the context of AD neuroimaging biomarkers. Studies in this category either evaluated the fairness of an existing algorithm in the context of sex-based effects or developed a model while using sex as a predictive variable. Machine learning frequently intersects with MRI data (21) and *APOE-ɛ4* carrier status (17). In only 3 studies, sex effects were not observed (Figure 2).

## Discussion

### Existence of sex differences in AD

In The Elephant in the Dark parable by the Persian poet Rumi, a group of people in a completely dark room attempt to determine what an elephant looks like. Each person touches a different part of the elephant’s body and forms their own idea based on that limited experience. One person, feeling the trunk, says the elephant is like a snake. Another, touching the ear, insists it is like a fan. Others, grasping the leg or the side, believe it resembles a tree trunk or a wall, respectively. This parable illustrates how an individual’s perceptions and beliefs can be limited and subjective, based on their own experiences. Each study in this review is one person feeling one aspect of the elephant, or one aspect of sex dependence in AD. This review aims to shed light on the elephant and reveal the underlying connections between each peer-reviewed study to enhance our understanding of sex differences in AD.

This systematic review of manuscripts demonstrates the clear existence of sex differences in the context of AD. Among 4555 manuscripts, only 2.6% (120) used sex as a biological predictive variable in the context of AD. Although this is a small percentage, these studies provide clear motivation for shifting from using biological sex as an adjusted variable to a predictive variable in analyses and design of future research protocols. More than 90% of the 120 manuscripts observed significant sex differences, while fewer than 10% observed no sex difference when using sex as a predictive variable in AD neuroimaging studies.

These 120 manuscripts investigated neuroimaging biomarkers in AD from various perspectives. Some studies investigated sex differences in terms of risk for AD, focusing on classical amyloid plaques, tau tangles, and neurodegeneration (A/T/N) imaging biomarker presentation (Table S10), while some focused on the clinical progression, discussing the notion of cognitive reserve (Table 1). Many studies examined the influence of factors such as hormones (Table S5), genetics (Table S6), inflammation and vascular health (Table S7), and lifestyle (physical activity, BMI, education; Table S8). Additionally, this review found that machine learning is an increasingly valuable tool in AD neuroimaging studies and has the potential to examine sex as a predictive variable, rather than an adjusted variable (Table S9).

### Sex differences in cognitive reserve: implications for AD progression

Cognitive reserve refers to the brain’s ability to maintain cognitive function despite the presence of AD pathology, such as amyloid and tau accumulation.^28^ Cognitive reserve is created mostly through life experiences, including education, occupational attainment, and engaging in mentally stimulating activities.^39^^,^^40^ While the concept of cognitive reserve is applicable to all individuals, this review suggests that biological sex plays a significant role in how cognitive reserve manifests and influences the progression of AD.^26^

An initial cognitive advantage is seen in verbal memory among females.^31^ Females with normal cognition show verbal learning and memory scores that are resilient to amyloid-beta-positive (Aβ+) status, determined by Aβ-PET,^26^ suggesting that they may possess a greater capacity to compensate for early AD-related brain changes.^26^ As the disease progresses to mild cognitive impairment (MCI), this cognitive reserve advantage diminishes, and Aβ+ females exhibit poorer verbal learning scores than their male counterparts.^26^ In contrast, males with Aβ-positive PET scans exhibit impaired verbal learning and memory scores regardless of cognitive status.^26^ This suggests a potential sex-specific neural reserve at the hippocampus, where female’s hippocampal integrity might be more resistant to early amyloid accumulation.^26^

There are sex differences in episodic memory, spatial memory, and language skills, where females with AD show better episodic memory, while males show better executive function as determined with the auditory verbal learning test and the digital scale, respectively.^38^ The sex differences in episodic memory and executive function align with sex differences in the strength of sensorimotor and attention networks, observed with fMRI.^38^ Females with AD pathology, as determined with CSF biomarkers and structural MRI, display fewer object-location-binding errors compared to their male counterparts across cognitive stages, which may be associated with a greater cognitive reserve in females.^36^ Sex differences in cognitive reserve can explain the variations across cognitive assessments in different AD stages.

Cognitive reserve is associated with education and bilingualism in both males and females with dementia. Higher education levels may moderate the effect of the *APOE-ɛ4* genotype, a significant AD risk factor, potentially acting as a cognitive buffer and delaying the accumulation of tau pathology via tau-PET, effectively delaying the onset of symptomatic AD.^28^ Bilingualism is also associated with increased cognitive reserve, leading to later onset of MCI. Bilingual individuals with MCI had more grey matter loss than monolinguals, as visualized with structural MRI.^24^ Interestingly, bilingual individuals with MCI converted to AD faster; this is consistent with the hypothesis that higher cognitive reserve leads to a more rapid decline once clinical symptoms manifest.^24^

Investigators of brain metabolic health using FDG-PET found that cognitively healthy females showed a significant metabolic brain age “youthful” advantage compared to males.^19^ However, this advantage disappeared among MCI individuals and AD patients, suggesting increased cognitive resilience to pathology in females.^19^ In contrast, another study found no significant differences in the cognitive evaluation Mini Mental State Examination between males and females in an early-onset AD cohort, despite females having a greater pathological burden as determined with Aβ and tau-PET imaging.^27^ These conflicting results highlight the need for further analysis of sex differences in cognitive evaluation scores.

Resting-state functional MRI suggests that sex moderates the impact of amyloid and *APOE-ɛ4* on the connectivity between the anterior and posterior default mode networks.^31^ Aβ+ females with an *APOE-ɛ4* allele show greater anterior to posterior default mode network connectivity, which correlates with better verbal learning, indicating a compensatory mechanism in females to maintain verbal learning in the face of AD risk factors.^31^

### Interplay between sex differences and other factors

#### Sex differences in imaging biomarkers

The major finding across several studies is that Aβ+ females demonstrate greater tau-PET retention than Aβ+ males, predominantly in the temporal lobe, but also extending to additional cortical regions.^13^^,^^41^^,^^42^ The biological mechanism for these sex differences is unclear; however, some studies reported associations with menopause and hormone replacement therapy (HRT). Most studies found sex differences in imaging biomarkers as a downstream effector caused by other factors such as genetic, lifestyle and/or the impact of hormonal changes.

#### The impact of hormonal changes on neuroimaging finding

Menopause is a major physiological transition in females’ midlife marked by depleted oestrogen levels.^15^ Oestrogenic deprivation in menopause is a known risk factor for AD in females.^43^ Neuroimaging studies also indicate that menopause is strongly associated with sex-dependent AD abnormalities in cognitively normal middle-aged participants.^44^ This association was more consistent and robust than other risk factors examined, such as obesity, hypertension, exercise, smoking status, or thyroid function.^44^ Midlife postmenopausal and perimenopausal females at high risk for AD exhibit faster rates of Aβ accumulation, reduced metabolism, and decreased hippocampal volume over a 2- to 3-year period.^44^^,^^45^ Imaging biomarker abnormalities, including MRI grey and white matter volumes, Aβ-PET, and FDG-PET, aligned along a gradient corresponding to menopausal stage, with progressively greater biomarker changes from premenopausal to perimenopausal to postmenopausal females, compared to age-matched males.^44^ This stepwise decline suggests that oestrogen declines are likely involved in observed AD biomarker abnormalities in females, potentially by coinciding with reduced brain bioenergetics.^44^ Together, perimenopausal and postmenopausal females have increased levels of Aβ^27^^,^^44^ and tau,^27^^,^^41^^,^^42^ decreased brain glucose metabolism,^27^^,^^44^^,^^46^ and reduced brain volumes^11^^,^^27^^,^^44^^,^^47–49^ compared to premenopausal females and age-matched males.

The impact of HRT or MHT on AD risk may be dependent upon *APOE* genotype.^15^  *APOE-ɛ4*-positive females who did not use MHT had a greater reduction in Aβ42/p-tau ratio over time, as compared to those who did.^47^ Further, when considering endogenous oestrogen levels in postmenopausal females, higher estrone (E1) and estradiol (E2) levels are associated with larger regional brain volumes, especially in females with lower Aβ42/40 ratios or lower Aβ42/p-tau ratios.^47^ This beneficial association is driven by non-*APOE-ɛ4* carriers, suggesting that *APOE-ɛ4* carriers might be less sensitive to the favourable effects of higher endogenous oestrogen levels on brain volume in postmenopause.^47^ Hysterectomy status also shows trends towards lower FDG uptake.^44^

These findings underscore the critical need to consider hormonal factors and endocrine ageing when studying sex differences in brain health and AD development.^27^^,^^49^

#### Genetics affecting AD pathology

The interaction between biological sex and *APOE* genetic variants significantly influences AD risk and progression, revealing distinct sex-specific patterns. Females carrying the *APOE-ɛ4* allele, the primary genetic risk factor for late-onset AD, exhibit a higher lifetime risk for AD and faster cognitive decline than males with the same genotype.^50^^,^^51^ Females exhibit stronger associations between *APOE-ɛ4* and AD biomarkers, like amyloid-beta and tau.^52^ Neuroimaging studies showed that *APOE-ɛ4*-positive females experience altered brain connectivity and elevated tau levels, compared to males, suggesting that sex modulates the allele’s neuropathological effects.^53^^,^^54^ In addition, the protective *APOE* ε2/ε3 allele reduces AD risk to a greater extent in females than in males.^55^ Hormonal influences further shape these differences, with postmenopausal oestrogen decline potentially increasing AD vulnerability in females by modifying APOE-related mechanisms,^56^ while testosterone in males may offer protective effects against *APOE-ɛ4*-related pathology.^57^ These findings underscore the importance of considering sex as a significant factor in understanding and addressing APOE-associated AD risk and progression.

#### Sex dependencies of inflammation and vascular health in AD

Sex differences significantly influence neuroinflammation and its association with AD pathology and symptoms. While direct imaging evidence of sex-specific neuroinflammation is limited, studies suggest females exhibit higher levels of inflammatory markers, like glial fibrillary acidic protein (GFAP), a biomarker for the glia cell type astrocytes that indicates reactive astrogliosis, and stronger links between neuroinflammation, neuropsychiatric symptoms, and brain atrophy. COVID-19 leads to increased neuroinflammation.^58^ During COVID-19 confinement, females had heightened anxiety and depression associated with AD risk factors.^59^ Additionally, GFAP-associated brain atrophy is reported in females.^60^ Similarly, a study investigating sex-specific trajectories of GFAP showed faster increases in plasma GFAP levels in females compared to males, indicating sex differences in inflammatory dynamics in preclinical AD stages.^61^ Conversely, males with sustained systemic inflammation have greater amyloid deposition.^62^ These findings highlight sex-specific inflammatory dynamics in AD progression influenced by immune function.

Sex differences in associations between imaging markers of neurovascular health and amyloid burden were also reported, with most studies reporting an association in males only or no sex-based effects.^20^^,^^63^^,^^64^ In a longitudinal analysis, higher numbers of vascular risk factors in midlife are associated with greater odds of amyloid positivity 20 years later in males, but not in females.^47^ However, females may show more severe imaging markers of white matter integrity and lesions in late-stage AD, which were associated with vascular risk factors in midlife.^20^

#### Lifestyle factors as AD risk factors

Overweight or obese individuals in preclinical AD show lower tau-PET and less cortical amyloid burden, compared to normal weight individuals.^45^ Being overweight or obese is also associated with better verbal memory in cognitively normal females, but worse global cognition among males, regardless of amyloid status.^45^ However, in a longitudinal study of non-demented individuals in birth cohorts from the United Kingdom, higher systolic and diastolic blood pressure are significantly associated with older brain age in females only.^46^ In females, higher BMI is associated with older brain age among *APOE-ɛ4* noncarriers and younger brain age among carriers, highlighting the relationship between genetic risk factors and obesity.

Studies investigated sex differences in adipose tissue compartments, such as visceral and subcutaneous adipose tissue, in relation to AD imaging. Visceral fat is more metabolically active and pro-inflammatory, while subcutaneous fat is less metabolically active. Diffusion basis spectrum imaging of white matter neuroinflammation and abdominal MRI were used to investigate the link between neuroinflammation and BMI, abdominal visceral adipose tissue, and subcutaneous adipose tissue in midlife.^48^ While visceral fat is associated with neuroinflammation in both males and females, subcutaneous fat and BMI are only associated with neuroinflammation in males.^48^ Additionally, higher visceral fat percentage is associated with higher and lower superior frontal gyrus volume in midlife males and females, respectively, while higher subcutaneous fat percentage is associated with lower middle frontal gyrus volume in males.^65^ This indicates that fat with different metabolic properties influences brain atrophy in a sex-specific manner.

Using an Aβ-PET tracer, a significant association is found between physical frailty and higher amyloid standardized uptake value ratio (SUVR)s in Aβ+ females.^2^ Lower levels of p-Tau-181 are linked to better nutritional status in females and maximal grip strength in males.^2^ Higher physical activity in midlife is related to lower entorhinal, inferior temporal, and rhinal cortex tau-PET SUVRs in males, while higher physical activity was related to higher rhinal cortex tau-PET SUVRs in females.^66^ There are no sex differences in the association between amyloid-PET SUVRs and physical activity. These findings indicate sex differences in the protective effects of physical activity for AD prevention.

In addition to BMI and physical activity, sex mediates the effects of sleep,^25^ C-reactive protein levels,^62^ education,^67^ air quality,^68^ and kidney function^16^ on cognition and AD progression. Several of these factors have inverse effects depending on sex, highlighting the need to consider lifestyle in treatment paradigms. While the contribution of lifestyle factors to AD is poorly understood, as humans are highly variable, it is imperative to account for sex and lifestyle differences in future AD research.

### Research avenues in the landscape of big data and machine learning

#### Machine learning

The application of machine learning techniques to neuroimaging data revealed several of the complex sex-specific patterns in AD pathophysiology and progression described above. For example, regarding cognitive reserve, machine learning methods found that cognitively healthy females exhibit significantly younger metabolic brain age than males, potentially reflecting hormonal neuroprotective effects that diminish postmenopause.^19^

Across papers relying on deep learning as a key method, sex emerges as a significant discriminative feature. Sex is the second most important predictor after *APOE-ɛ4* status when detecting preclinical AD,^6^ and sex is the only significant demographic predictor when differentiating AD from frontotemporal dementia.^69^ As the use of machine learning increases, it is important to consider algorithmic fairness, which involves the absence of prejudice or favouritism towards any individual or group based on inherent characteristics such as sex, race, or ethnicity. A fair algorithm yields comparable predictive performance, particularly sensitivity, across protected groups and satisfies principles such as equal opportunity (equal true-positive rates across groups) and equalized odds.

Current models exhibit varying sensitivities between males and females, particularly during transitions from normal cognition to MCI.^70^

Machine learning is also pivotal to identifying the intersection between geographical and socioeconomic factors and sex differences; there are significant female brain-age gaps in regions with greater gender inequality.^71^ Similarly, deep learning algorithms aided researchers in identifying sex-specific associations between cardiometabolic risk and white matter ageing. Males show greater vulnerability to early cardiometabolic effects, while older females exhibit unique patterns potentially indicating frailty-related neurodegeneration.^49^

Machine learning is becoming a fruitful tool in analysing neuroimaging datasets, which is a critical part of AD diagnosis, treatment, and research. Unfortunately, these findings cannot be generalized to everyone with AD until the data acquired is representative of the population and is easily accessible. The studies included in this review provided incomplete racial demographics, but the majority of the studies (100/120) originate from countries with a majority white, non-Hispanic population, likely indicating the findings from this review are more applicable to white, non-Hispanic populations (Tables S3 and S4) and may not be representative of global demographics. This systematic review includes only 2 multi-country/global analyses. One considers 9758 participants enrolled in phase III clinical trial from 29 countries across North America, South America, Western Europe, Oceania, Eastern Europe, Asia, and Africa.^72^ In this study, the only country in Africa is South Africa, making up <1% of total participants, and the racial demographics of these participants were not noted.^72^ Further, the first authors of the articles included in this review are not from institutions located in South America (Figure 4). The other multi-country analysis studied participants in South America and used machine learning to compare sex differences in brain age between Latin American and Caribbean (LAC) countries and non-LAC countries.^71^ There is a stark absence of studies from African and South American countries (see Table S2) Without funding to encourage cross-country collaboration and neuroimaging studies in under-resourced regions, particularly those without access to scanners, the imaging field risks perpetuating inequities and limiting the accuracy of global analyses.^71^ Researchers must adjust neuroimaging recruitment efforts and increase cross-country collaborations to increase the scientific validity of the work presented and conduct scientifically rigorous analyses.

#### Data sharing

Data sharing is becoming increasingly available and valuable, but barriers to open access data remain, including data requests, which vary in length and complexity. Recent advances in informative data science have increased the ease of large data analyses; the field should take advantage of this to strengthen findings. For example, cohorts in studies included in this review were 57% female, on average. These studies varied in size, with smaller analyses having only 40 participants in the study. Humans are extremely variable, and smaller projects are not well powered to find statistically significant sex differences. Increasing accessibility of data will enable institutions with limited imaging capabilities to conduct large analyses and find statistically significant differences between sexes. Additionally, increasing data sharing is advantageous for improving patient care and outcomes. Data collection via imaging, CSF, and plasma is extremely strenuous, especially for the targeted population with MCI or AD due to frequently limited means of transportation, and the circumstances of data collection may induce stress. Thus, it is critical that we thoroughly analyse the data collected. Open access to data is critical to the pursuit of a cure for AD that is applicable to the entire population.

We collected information on available data centres, which can be found in Tables S3 and S4. These tables detail methods of accessing data from different centres, including data request requirements.

## Limitation and bias

There are noteworthy issues that limit the rigour of this review. First, we excluded elegant studies with large cohorts that would be well powered to examine statistically significant sex differences as they consider sex as an adjusted variable, not a biological predictive variable. Second, although more than 6000 papers were screened, it is likely that some studies were missed, evidenced by the fact that 12 papers that met the inclusion criteria were manually added. Third, inappropriate exclusion may have occurred during abstract review. If sex difference was a secondary analysis, meaning they specifically mentioned sex as an adjusted variable in the abstract but used sex as a predictive variable in further analyses, the study was excluded. Finally, missing data in this review may arise from a lack of negative data reported; if papers did not find a significant difference, they may have elected not to report the sex analysis.

## Conclusions

This review demonstrated that a vast majority of studies that assessed sex effects via neuroimaging biomarkers in AD found sex-related differences. These studies used a variety of neuroimaging tools, including MRI, amyloid-PET, tau-PET, and FDG-PET, and found sex-related differences in cognitive reserve, hormones, *APOE-ɛ4* genotype, inflammation, vascular health, and lifestyle factors. Machine learning studies improved algorithmic performance when incorporating sex effects and served as a useful tool for predicting disease progression.

The authors of this review implore investigators in this field to consider biological sex when exploring neuroimaging biomarkers and AD progression and encourage data sharing. This review highlights the importance of considering biological sex in AD to enhance diagnostic accuracy, tailor therapeutics, and improve the health of all individuals affected by AD.

## Supplementary Material

tqag011_Supplementary_Data
