# Supplementary material for: Sex differences in Alzheimer’s disease: a systematic review of two decades of neuroimaging research
Source: Br J Radiol. 2026 Jan 16;99(1180):702–13. doi: 10.1093/bjr/tqag011 (PMC13070646; doi:10.1093/bjr/tqag011)
Supplement: tqag011_Supplementary_Data [file tqag011_supplementary_data.zip › Table S2.docx]

**Table S2:** Countries of residence of first authors' institutions investigating sex difference in AD. The United States contributed the most with 56 studies, followed by China with 11 studies.

|  | First Author’s Institution location** | # Manuscript |
| --- | --- | --- |
| 1 | USA | 56 |
| 2 | China | 11 |
| 3 | Australia | 8 |
| 4 | Canada | 8 |
| 5 | United Kingdom | 8 |
| 6 | Germany | 6 |
| 7 | Republic of Korea | 5 |
| 8 | Sweden | 5 |
| 9 | Netherland | 4 |
| 10 | Norway | 3 |
| 11 | Italy | 2 |
| 12 | Japan | 2 |
| 13 | Switzerland | 2 |
| 14 | Belgium | 1 |
| 15 | Poland | 1 |
| 16 | India | 1 |
| 17 | Portugal | 1 |
| 18 | Taiwan | 1 |

** 20 manuscripts have co-authors from multiple countries. 26 manuscripts have co-authors from Europe (17 manuscripts from individual countries and 9 manuscripts from collaborations between European countries). 1 manuscript includes authors from 15 countries (7 Latin American and Caribbean countries and 8 non-LAC countries), another manuscript included participants and investigators from 29 countries.
