# Supplementary material for: Sex differences in Alzheimer’s disease: a systematic review of two decades of neuroimaging research
Source: Br J Radiol. 2026 Jan 16;99(1180):702–13. doi: 10.1093/bjr/tqag011 (PMC13070646; doi:10.1093/bjr/tqag011)
Supplement: tqag011_Supplementary_Data [file tqag011_supplementary_data.zip › Appendix S1 - Systematic Review Search Methods.docx]

# Appendix S1: Systematic Review Search Methods for Systematic Review

The material contained in this document was created and prepared for the Systematic Review search strategy using the Library Systematic Review request form. All search strategies and written methodology were created using the standards and guidelines for conducting and reporting systematic reviews set forth by the Preferred Reporting Items for Systematic Reviews and Meta-Analyses [(PRISMA),](http://www.prisma-statement.org/) the National Academies (IOM) [Standards for Systematic Reviews](http://www.nationalacademies.org/hmd/Reports/2011/Finding-What-Works-in-Health-Care-Standards-for-Systematic-Reviews/Standards.aspx), the [Cochrane Handbook of Systematic reviews](http://training.cochrane.org/handbook), and Peer Review of Systematic Search Strategies [(PRESS)](https://www.cadth.ca/resources/finding-evidence/press). In accordance with these guidelines, the following items are included in this document:

- Fully reproducible search strategies for each database searched.
- A written methodology section explaining how the search was created and designed.

## Methodology Section

Date: 11/25/2024

A medical librarian (LHY) searched the literature for records including the concepts of Alzheimer disease, dementia, biological sex differences, neuroimaging between 2004-2024 removing animal studies where possible. The librarian created search strategies using a combination of keywords and controlled vocabulary in Embase.com 1947- , Ovid Medline 1946- , Scopus 1823- , Cochrane Central Register of Controlled Trials (CENTRAL), The Cochrane Databases of Systematic Reviews (CDSR), APA PsycInfo 1800s- , and Clinicaltrials.gov 1997- . All search strategies were completed November 25, 2024, limited to 2004-2024 and a total of 6,094 results were found. 1,546 duplicate records were deleted using Covidence (Veritas Health Innovation, Melbourne, Australia) resulting in a total of 4,548 unique citations included in the project library.

Fully reproducible search strategies for each database are as follow:

Embase
Date Searched: 11/25/2024

Applied Database Supplied Limits: 2004-2025

Number of Results: 3,706

Full Search Strategy:

(('Alzheimer disease'/exp OR (Alzeimer* OR Alzheimer* OR ‘diffuse cortical sclerosis’):ti,ab,kw) AND ('biological sex'/exp OR 'female'/de OR 'gender'/de OR 'gender and sex'/de OR 'male'/de OR 'sex'/de OR 'sex difference'/de OR 'sex factor'/de OR 'sexual characteristics'/de OR (sex OR female OR females OR woman OR women OR gender OR male OR males OR man OR men OR ‘sex-dependent’ OR ‘sex-related’ OR ‘sex-specific’):ti,ab,kw OR (sexual NEAR/2 (difference* OR characteristic*)):ti,ab,kw) AND ('diagnostic imaging'/de OR 'imaging'/de OR 'neuroimaging'/exp OR 'nuclear magnetic resonance imaging'/de OR 'positron emission tomography'/de OR 'positron emission tomography-computed tomography'/de OR (neuroimage* OR ‘Amyloid-Related Imaging Abnormal*’ OR ARIA OR ‘ophthalmo diaphanoscopy’ OR ‘diffusion weighted magnetic resonance’ OR ‘diffusion weighted nuclear magnetic resonance’ OR MRI OR fMRI OR ‘R-fMRI’ OR ‘rsfMRI’ OR zeugmatography OR ‘NMR tomography’ OR ‘MR Tomography’ OR ‘Proton Spin Tomography’ OR imaging OR MRI OR ‘magnetic resonance tomography’ OR ‘neuro-imaging’ OR neuroimaging OR neuropsychologic* OR ‘positron emission tomography’ OR PET OR ‘positron tomograph*’ OR ‘positron-emission tomograph*’ OR ‘positron emission tomograph*’ OR ‘tauopathy-PET’):ti,ab,kw) AND ('equity'/exp OR 'health care disparity'/exp OR 'health equity'/exp OR 'health care need'/exp OR 'economic inequality'/exp OR 'gender inequality'/exp OR 'lowest income group'/exp OR 'working poor'/exp OR 'middle income group'/exp OR 'social determinants of health'/exp OR 'race'/exp OR 'ethnic group'/exp OR 'racial disparity'/exp OR 'ethnicity'/exp OR 'ethnic difference'/exp OR 'socioeconomics'/exp OR 'social status'/exp OR 'ancestry group'/exp OR (equity OR disparit* OR ‘health care need’ OR 'health care demand' OR 'healthcare demand' OR 'healthcare need' OR inequit* OR inequality* OR ‘lowest income’ OR ‘low income’ OR ‘low-income’ OR ‘middle income' OR ‘LMIC*’ OR ‘social determinants’ OR race OR racial OR ‘Continental Population Groups’ OR 'ethno-linguistic group' OR 'ethnolinguistic group' OR Nationality OR Nationalities OR Ethnicity OR 'racial-ethnic' OR 'race-ethnicity' OR 'economic value of life' OR 'indigent health care' OR 'medical indigency' OR 'social economic' OR 'social economics' OR 'social-economic' OR 'socio-economic' OR 'socio-economics' OR socioeconomic OR 'value of life'):ti,ab,kw OR ((ethnic OR race OR racial) NEAR/2 (group* OR minorit* OR origin* OR population* OR status OR difference* OR disparit*)):ti,ab,kw OR ((ethnic OR ethnical OR ethnologic) NEAR/2 (distribution* OR variation* OR difference*)):ti,ab,kw OR (social NEAR/2 (achievement* OR condition* OR status OR employment OR function OR identit* OR importance OR rank* OR standing* OR state*)):ti,ab,kw OR (('continental ancestry' OR ‘continental population' OR racial) NEAR/2 (group*)):ti,ab,kw)) NOT ('animal'/exp NOT ('animal'/exp AND 'human'/exp)) AND [2004-2024]/py

Ovid Medline
Date Searched: 11/25/2024
Applied Database Supplied Limits: 2004-2024
Number of Results: 782

Full Search Strategy:

((exp Alzheimer Disease/ OR (Alzeimer* OR Alzheimer* OR diffuse cortical sclerosis).ti,ab,kf.) AND (Sex Characteristics/ or Sex Factors/ OR Female/ OR Male/ OR Sex/ OR (sex OR female OR females OR woman OR women OR gender OR male OR males OR man OR men OR sex-dependent OR sex-related OR sex-specific).ti,ab,kf. OR (sexual ADJ2 (difference* OR characteristic*)).ti,ab,kf.) AND (Diagnostic Imaging/ OR exp Neuroimaging/ OR Magnetic Resonance Imaging/ OR Positron-Emission Tomography/ OR Positron Emission Tomography Computed Tomography/ OR (neuroimage* OR Amyloid-Related Imaging Abnormal* OR ARIA OR ophthalmo diaphanoscopy OR diffusion weighted magnetic resonance OR diffusion weighted nuclear magnetic resonance OR MRI OR fMRI OR R-fMRI OR rsfMRI OR zeugmatography OR NMR tomography OR MR Tomography OR Proton Spin Tomography OR imaging OR MRI OR magnetic resonance tomography OR neuro-imaging OR neuroimaging OR neuropsychologic* OR positron emission tomography OR PET OR positron tomograph* OR positron-emission tomograph* OR positron emission tomograph* OR tauopathy-PET).ti,ab,kf.) AND (exp Health Equity/ OR exp Healthcare Disparities/ OR exp "Health Services Needs and Demand"/ OR exp Socioeconomic Factors/ OR exp Health Status Disparities/ OR exp Gender Equity/ OR exp Working Poor/ OR exp Poverty/ OR exp "Social Determinants of Health"/ OR exp Racial Groups/ OR exp Ethnicity/ OR exp Health Status Disparities/ OR exp Social Status/ OR (equity OR disparit* OR health care need OR health care demand OR healthcare demand OR healthcare need OR inequit* OR inequality* OR lowest income OR low income OR low-income OR middle income OR LMIC* OR social determinants OR race OR racial OR Continental Population Groups OR ethno-linguistic group OR ethnolinguistic group OR Nationality OR Nationalities OR Ethnicity OR racial-ethnic OR race-ethnicity OR economic value of life OR indigent health care OR medical indigency OR social economic OR social economics OR social-economic OR socio-economic OR socio-economics OR socioeconomic OR value of life).ti,ab,kf. OR ((ethnic OR race OR racial) ADJ2 (group* OR minorit* OR origin* OR population* OR status OR difference* OR disparit*)).ti,ab,kf. OR ((ethnic OR ethnical OR ethnologic) ADJ2 (distribution* OR variation* OR difference*)).ti,ab,kf. OR (social ADJ2 (achievement* OR condition* OR status OR employment OR function OR identit* OR importance OR rank* OR standing* OR state*)).ti,ab,kf. OR ((continental ancestry OR continental population OR racial) ADJ2 (group*)).ti,ab,kf.)) NOT (exp Animals/ NOT (exp Animals/ AND exp Humans/))

Scopus
Date Searched: 11/25/2024
Applied Database Supplied Limits: 2004-2024
Number of Results: 1,356

Full Search Strategy:

(((TITLE-ABS-KEY(Alzeimer* OR Alzheimer* OR “diffuse cortical sclerosis”))) AND ((TITLE-ABS-KEY(sex OR female OR females OR woman OR women OR gender OR male OR males OR man OR men OR “sex-dependent” OR “sex-related” OR “sex-specific”)) OR (TITLE-ABS-KEY(sexual W/2 (difference* OR characteristic*)))) AND ((TITLE-ABS-KEY(neuroimage* OR “Amyloid-Related Imaging Abnormal*” OR ARIA OR “ophthalmo diaphanoscopy” OR “diffusion weighted magnetic resonance” OR “diffusion weighted nuclear magnetic resonance” OR MRI OR fMRI OR “R-fMRI” OR “rsfMRI” OR zeugmatography OR “NMR tomography” OR “MR Tomography” OR “Proton Spin Tomography” OR imaging OR MRI OR “magnetic resonance tomography” OR “neuro-imaging” OR neuroimaging OR neuropsychologic* OR “positron emission tomography” OR PET OR “positron tomograph*” OR “positron-emission tomograph*” OR “positron emission tomograph*” OR “tauopathy-PET”))) AND ((TITLE-ABS-KEY(equity OR disparit* OR “health care need” OR “health care demand” OR “healthcare demand” OR “healthcare need” OR inequit* OR inequality* OR “lowest income” OR “low income” OR “low-income” OR “middle income” OR “LMIC*” OR “social determinants” OR race OR racial OR “Continental Population Groups” OR “ethno-linguistic group” OR “ethnolinguistic group” OR Nationality OR Nationalities OR Ethnicity OR “racial-ethnic” OR “race-ethnicity” OR “economic value of life” OR “indigent health care” OR “medical indigency” OR “social economic” OR “social economics” OR “social-economic” OR “socio-economic” OR “socio-economics” OR socioeconomic OR “value of life”)) OR (TITLE-ABS-KEY((ethnic OR race OR racial) W/2 (group* OR minorit* OR origin* OR population* OR status OR difference* OR disparit*))) OR (TITLE-ABS-KEY((ethnic OR ethnical OR ethnologic) W/2 (distribution* OR variation* OR difference*))) OR (TITLE-ABS-KEY(social W/2 (achievement* OR condition* OR status OR employment OR function OR identit* OR importance OR rank* OR standing* OR state*))) OR (TITLE-ABS-KEY((“continental ancestry” OR “continental population” OR racial) W/2 (group*))))) AND NOT (((TITLE(animal*)) AND PUBYEAR > 2003 AND PUBYEAR < 2025

### The Cochrane Library

Date Searched: 11/25/2024
Applied Database Supplied Limits: 2004-2024
Number of Results

CENTRAL: 85

CDSR: 0

Full Search Strategy:

(([mh “Alzheimer Disease”] OR (Alzeimer* OR Alzheimer* OR “diffuse cortical sclerosis”):ti,ab,kw) AND ([mh “Sex Characteristics”] or [mh “Sex Factors”] OR [mh “Female”] OR [mh “Male”] OR [mh “Sex”] OR (sex OR female OR females OR woman OR women OR gender OR male OR males OR man OR men OR “sex dependent” OR “sex related” OR “sex specific”):ti,ab,kw OR (sexual NEAR/2 (difference* OR characteristic*)):ti,ab,kw) AND ([mh “Diagnostic Imaging”] OR [mh “Neuroimaging”] OR [mh “Magnetic Resonance Imaging”] OR [mh “Positron-Emission Tomography”] OR [mh “Positron Emission Tomography Computed Tomography”] OR (neuroimage* OR “Amyloid Related Imaging Abnormal*” OR ARIA OR “ophthalmo diaphanoscopy” OR “diffusion weighted magnetic resonance” OR “diffusion weighted nuclear magnetic resonance” OR MRI OR fMRI OR “R fMRI” OR “rsfMRI” OR zeugmatography OR “NMR tomography” OR “MR Tomography” OR “Proton Spin Tomography” OR imaging OR MRI OR “magnetic resonance tomography” OR “neuro imaging” OR neuroimaging OR neuropsychologic* OR “positron emission tomography” OR PET OR “positron tomograph*” OR “positron emission tomograph*” OR “positron emission tomograph*” OR “tauopathy PET”):ti,ab,kw) AND ([mh “Health Equity”] OR [mh “Healthcare Disparities”] OR [mh “Health Services Needs and Demand”] OR [mh “Socioeconomic Factors”] OR [mh “Health Status Disparities”] OR [mh “Gender Equity”] OR [mh “Working Poor”] OR [mh “Poverty”] OR [mh “Social Determinants of Health”] OR [mh “Racial Groups”] OR [mh “Ethnicity”] OR [mh “Health Status Disparities”] OR [mh “Social Status”] OR (equity OR disparit* OR “health care need” OR “health care demand” OR “healthcare demand” OR “healthcare need” OR inequit* OR inequality* OR “lowest income” OR “low income” OR “low income” OR “middle income” OR “LMIC*” OR “social determinants” OR race OR racial OR “Continental Population Groups” OR “ethno linguistic group” OR “ethnolinguistic group” OR Nationality OR Nationalities OR Ethnicity OR “racial ethnic” OR “race ethnicity” OR “economic value of life” OR “indigent health care” OR “medical indigency” OR “social economic” OR “social economics” OR “social economic” OR “socio economic” OR “socio economics” OR socioeconomic OR “value of life”):ti,ab,kw OR ((ethnic OR race OR racial) NEAR/2 (group* OR minorit* OR origin* OR population* OR status OR difference* OR disparit*)):ti,ab,kw OR ((ethnic OR ethnical OR ethnologic) NEAR/2 (distribution* OR variation* OR difference*)):ti,ab,kw OR (social NEAR/2 (achievement* OR condition* OR status OR employment OR function OR identit* OR importance OR rank* OR standing* OR state*)):ti,ab,kw OR ((“continental ancestry” OR “continental population” OR racial) NEAR/2 (group*)):ti,ab,kw)) NOT ([mh “Animals”] NOT ([mh “Animals”] AND [mh “Humans”]))

APA PsycInfo
Date Searched: 11/25/2024
Applied Database Supplied Limits: 2004-2024
Number of Results: 161

Full Search Strategy:

1. (DE "Alzheimer's Disease" OR TI (Alzeimer* OR Alzheimer* OR “diffuse cortical sclerosis”) OR AB (Alzeimer* OR Alzheimer* OR “diffuse cortical sclerosis”))

AND

1. ((DE "Sex") AND (DE "Human Sex Differences" OR DE "Human Males" OR DE "Human Females") OR TI (sex OR female OR females OR woman OR women OR gender OR male OR males OR man OR men OR “sex-dependent” OR “sex-related” OR “sex-specific”) OR AB (sex OR female OR females OR woman OR women OR gender OR male OR males OR man OR men OR “sex-dependent” OR “sex-related” OR “sex-specific”) OR TI (sexual N2 (difference* OR characteristic*)) OR AB (sexual N2 (difference* OR characteristic*)))

AND

1. (DE "Medical Imaging" OR DE "Magnetic Resonance Imaging" OR DE "Neuroimaging" OR DE "Positron Emission Tomography" OR TI (neuroimage* OR “Amyloid-Related Imaging Abnormal*” OR ARIA OR “ophthalmo diaphanoscopy” OR “diffusion weighted magnetic resonance” OR “diffusion weighted nuclear magnetic resonance” OR MRI OR fMRI OR “R-fMRI” OR “rsfMRI” OR zeugmatography OR “NMR tomography” OR “MR Tomography” OR “Proton Spin Tomography” OR imaging OR MRI OR “magnetic resonance tomography” OR “neuro-imaging” OR neuroimaging OR neuropsychologic* OR “positron emission tomography” OR PET OR “positron tomograph*” OR “positron-emission tomograph*” OR “positron emission tomograph*” OR “tauopathy-PET”) OR AB (neuroimage* OR “Amyloid-Related Imaging Abnormal*” OR ARIA OR “ophthalmo diaphanoscopy” OR “diffusion weighted magnetic resonance” OR “diffusion weighted nuclear magnetic resonance” OR MRI OR fMRI OR “R-fMRI” OR “rsfMRI” OR zeugmatography OR “NMR tomography” OR “MR Tomography” OR “Proton Spin Tomography” OR imaging OR MRI OR “magnetic resonance tomography” OR “neuro-imaging” OR neuroimaging OR neuropsychologic* OR “positron emission tomography” OR PET OR “positron tomograph*” OR “positron-emission tomograph*” OR “positron emission tomograph*” OR “tauopathy-PET”))

AND

1. (DE "Economic Inequality" OR DE "Equity" OR DE "Health Disparities" OR DE "Income Economic" OR DE "Income Level" OR DE "Lower Income Level" OR DE "Lower Socioeconomic Status" OR DE "Medically Underserved Communities" OR DE "Middle Income Level" OR DE "Race and Ethnic Discrimination" OR DE "Racial and Ethnic Differences" OR DE "Racial and Ethnic Groups" OR DE "Racial Disparities" OR DE "Social Determinants of Health" OR DE "Social Equity" OR DE "Social Status" OR DE "Socioeconomic Disparities" OR DE "Socioeconomic Status" OR TI (equity OR disparit* OR “health care need” OR “health care demand” OR “healthcare demand” OR “healthcare need” OR inequit* OR inequality* OR “lowest income” OR “low income” OR “low-income” OR “middle income” OR “LMIC*” OR “social determinants” OR race OR racial OR “Continental Population Groups” OR “ethno-linguistic group” OR “ethnolinguistic group” OR Nationality OR Nationalities OR Ethnicity OR “racial-ethnic” OR “race-ethnicity” OR “economic value of life” OR “indigent health care” OR “medical indigency” OR “social economic” OR “social economics” OR “social-economic” OR “socio-economic” OR “socio-economics” OR socioeconomic OR “value of life”) OR AB (equity OR disparit* OR “health care need” OR “health care demand” OR “healthcare demand” OR “healthcare need” OR inequit* OR inequality* OR “lowest income” OR “low income” OR “low-income” OR “middle income” OR “LMIC*” OR “social determinants” OR race OR racial OR “Continental Population Groups” OR “ethno-linguistic group” OR “ethnolinguistic group” OR Nationality OR Nationalities OR Ethnicity OR “racial-ethnic” OR “race-ethnicity” OR “economic value of life” OR “indigent health care” OR “medical indigency” OR “social economic” OR “social economics” OR “social-economic” OR “socio-economic” OR “socio-economics” OR socioeconomic OR “value of life”) OR TI ((ethnic OR race OR racial) N2 (group* OR minorit* OR origin* OR population* OR status OR difference* OR disparit*)) OR AB ((ethnic OR race OR racial) N2 (group* OR minorit* OR origin* OR population* OR status OR difference* OR disparit*)) OR TI ((ethnic OR ethnical OR ethnologic) N2 (distribution* OR variation* OR difference*)) OR AB ((ethnic OR ethnical OR ethnologic) N2 (distribution* OR variation* OR difference*)) OR TI (social N2 (achievement* OR condition* OR status OR employment OR function OR identit* OR importance OR rank* OR standing* OR state*)) OR AB (social N2 (achievement* OR condition* OR status OR employment OR function OR identit* OR importance OR rank* OR standing* OR state*)) OR TI ((“continental ancestry” OR “continental population” OR racial) N2 (group*)) OR AB ((“continental ancestry” OR “continental population” OR racial) N2 (group*)))

NOT

1. ((DE "Animals" NOT (DE "Animals" AND (DE "Human Males" OR DE "Human Females"))))

ClinicalTrials.gov
Date Searched: 11/25/2024

Number of Results: 4

Full Search Strategy:

Condition/Disease: (Alzeimer* OR Alzheimer*)

Other terms: (“biological sex” OR “sex difference*”) OR (equity OR disparities OR race OR socioeconomic)

Intervention/treatment: (neuroimaging OR MRI OR PET)
